# Persistent Symptoms and Health Needs of Women and Men With Non-Obstructed Coronary Arteries in the Years Following Coronary Angiography

**DOI:** 10.3389/fcvm.2021.670843

**Published:** 2021-05-03

**Authors:** Floor Groepenhoff, Anouk L. M. Eikendal, Z. H. Saskia Rittersma, Crystel M. Gijsberts, Folkert W. Asselbergs, Imo E. Hoefer, Gerard Pasterkamp, Frans H. Rutten, N. Charlotte Onland-Moret, Hester M. Den Ruijter

**Affiliations:** ^1^Laboratory of Experimental Cardiology, University Medical Center Utrecht, Utrecht University, Utrecht, Netherlands; ^2^Central Diagnostic Laboratory, University Medical Center Utrecht, Utrecht University, Utrecht, Netherlands; ^3^Department of Cardiology, Division Heart and Lungs, University Medical Centre Utrecht, Utrecht University, Utrecht, Netherlands; ^4^Institute of Cardiovascular Science, Faculty of Population Health Sciences, University College London, London, United Kingdom; ^5^Institute of Health Informatics, Faculty of Population Health Sciences, University College London, London, United Kingdom; ^6^Department of General Practice, Julius Center for Health Sciences and Primary Care, University Medical Center Utrecht, Utrecht University, Utrecht, Netherlands; ^7^Julius Center for Health Sciences and Primary Care, University Medical Center Utrecht, Utrecht University, Utrecht, Netherlands

**Keywords:** coronary angiography, primary care, healthcare consumption, prolonged anginal complaints, non-obstructed coronary arteries

## Abstract

**Background:** The prognosis of women and men with persistent anginal complaints and non-obstructed coronary arteries is impaired as compared with asymptomatic women and men. The increased healthcare burden in the hospital due to repeated coronary angiography in these women and men has been documented, yet little is known about the percentage of women and men who remain symptomatic and under care of the general practitioner in the years following a coronary angiographic outcome of non-obstructed coronary arteries.

**Methods:** From the Utrecht Coronary Biobank study, including individuals who underwent a coronary angiography from 2011 to 2015 (*N* = 2,546, 27% women), we selected women and men with non-obstructed coronary arteries (*N* = 687, 39% women). This population was linked to the Julius General Practitioners Network (JGPN); a database with routine care data of general practitioners. For every individual with non-obstructed coronary arteries, we selected an asymptomatic non-referred age-, sex-, and general practitioner-matched individual from the JGPN. We compared the healthcare consumption of men and women with non-obstructed coronary arteries to these matched individuals. The McNemar's test was used for pairwise comparison, and sex differences were assessed using stratified analyses.

**Results:** The prevalence of non-obstructed coronary arteries was higher in women as compared with men (39 vs. 23%). During a median follow-up of 7 years [IQR 6.4–8.0], 89% of the individuals with non-obstructed coronary arteries (91% women and 87% men) visited their general practitioner for one or more cardiovascular consultations. This was compared to 34% of the matched individuals (89 vs. 34%, *p* < 0.001). The consultations were most often for angina (equivalents) (57 vs. 11%, *p* < 0.001) and heart failure (10 vs. 2%, *p* = 0.015). In addition, they more often consulted the general practitioner for psychosocial complaints (31 vs. 15%, *p* = 0.005). Findings were similar for women and men.

**Conclusions:** A coronary angiographic outcome of non-obstructed coronary arteries is more common in women than in men. In the years following the coronary angiography, the majority of the population remains symptomatic. Both women and men with non-obstructed coronary arteries had higher health needs for angina, heart failure, and psychosocial complaints than matched asymptomatic individuals.

## Introduction

Women with persistent complaints with non-obstructed coronary arteries have a poor prognosis (1). Recent evidence points to alternative mechanisms of cardiac ischemia that are often not detected during coronary angiography. Vasospastic angina and coronary microvascular disease may explain persistent symptoms, and the predisposition for heart failure with preserved ejection fraction (HFpEF) (2–4).

Relatively more women than men undergoing coronary angiography are found to have non-obstructed coronary arteries (5). Specifically, the percentage of women undergoing coronary angiography that are referred back to the general practitioner with the outcome non-obstructed coronary arteries is even double the percentage of men (6). As such, non-obstructed coronary arteries is mainly considered a “women's problem” (5) and studies have been performed mostly in women (7) hampering a comparison with men (8). Furthermore, most studies focused selectively on women with persisting symptoms, not on all women with non-obstructed coronary arteries, that is, also including those without persisting anginal complaints (1, 7).

Prior studies on the outcome of these women were mainly investigating major adverse cardiovascular events as registered in the hospital setting (9, 10). In the Women's Ischemia Syndrome Evaluation (WISE) study, over 15% of women with non-obstructed coronary arteries underwent a second coronary angiography during 5 years of follow up (11). An European study evaluating both women and men undergoing coronary angiography with non-obstructed coronary arteries showed similar results in both sexes, namely more hospitalizations, repeat coronary angiography, and overall more consultations in primary care as compared with asymptomatic individuals from the general population (12). No information was available on the reasons for these consultations in primary care. Thus, even though women and men with non-obstructed coronary arteries may not experience a hard endpoint, they may suffer from persistent symptoms and associated morbidities that affect health-related quality of life (11). In this study we assessed the percentage of women and men with non-obstructed coronary arteries who remain symptomatic in the years following the coronary angiography by linkage to a general practitioner's registry. In addition, we assessed their health need by comparing all cardiovascular and psychosocial primary care consultations to matched asymptomatic individuals.

## Materials and Methods

### Patient Population

**Figure 1:**
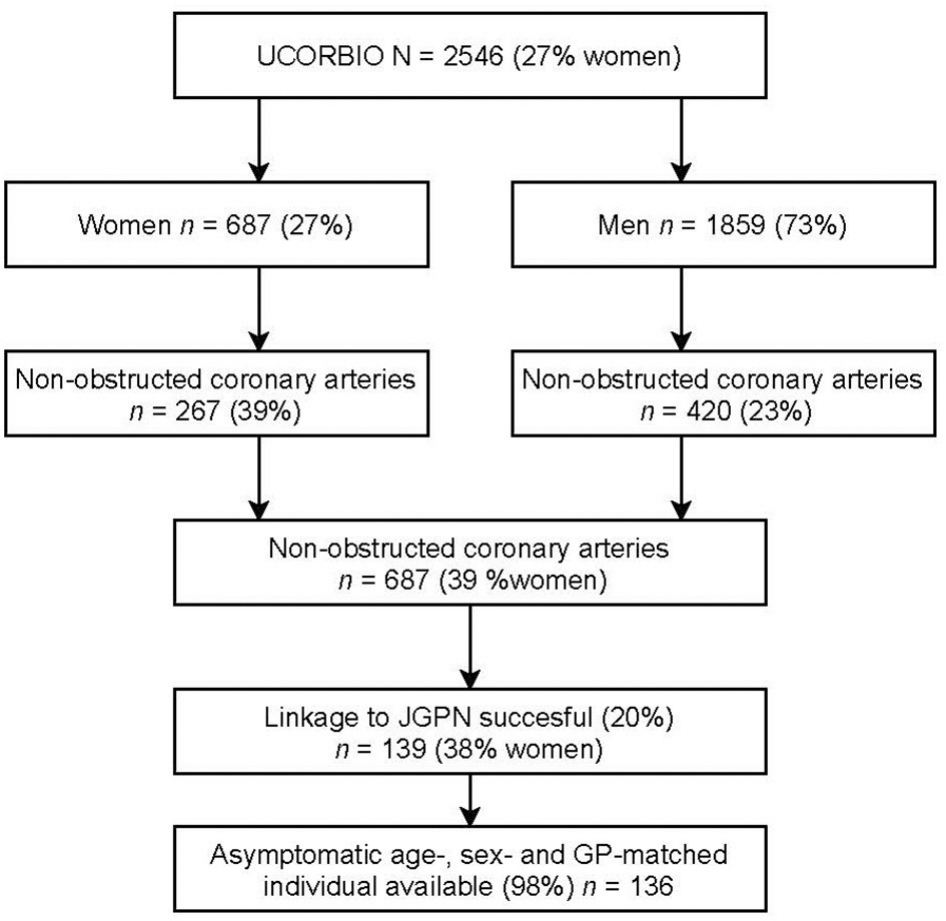
Flowchart of selection of the study population.

The UCORBIO cohort has been extensively described elsewhere (8). In short, UCORBIO comprises 2,546 consecutive routine care participants that underwent a coronary angiography between 2011 and 2015 at the UMC Utrecht. The study was approved by the Medical Ethics Committee of the UMC Utrecht (reference number 11–183), all participants provided written informed consent and the study confirmed to the Declaration of Helsinki. Standardized electronic case record files were completed at baseline (containing age, sex, cardiovascular risk factors, indication for and findings from coronary angiography, medication use, treatment initiated after coronary angiography). Significant coronary artery disease was defined as >50% stenosis, non-obstructive coronary artery disease as <50% stenosis and/or wall irregularities and no coronary artery disease as normal coronary arteries. For this study we only analyzed data of women and men with non-obstructed coronary arteries consisting of the group of no and non-obstructed coronary arteries [*n* = 687; 267 (39% females)] who consented to linkage of their data (Figure 1).

Follow up information on persisting symptoms and primary care consultations was obtained by linkage to the Julius General Practitioners Network (JGPN) database (13). Linkage was performed by a trusted third party using anonymous pseudo-identification to ensure protection of the privacy of the patients during linkage. The JGPN database comprises routine care data of individuals registered with participating general practitioners from the vicinity of Utrecht, The Netherlands. Because the catchment area for the UCORBIO patients was larger than the vicinity of Utrecht, only 139 (20%) of the 687 UCORBIO patients could be identified within the JGPN database. We restricted our analyses to these 139 patients (*n* = 53 (38%) women). Those identified within the JGPN database did not significantly differ from the remaining UCORBIO patients that could not be linked to JGPN (Supplementary Table 1). Follow-up information was available for a median of 7 years [IQR, 6.4–8.0].

### Comparison Population

The JGPN is a representative sample of the Dutch population; all Dutch citizens are registered with a general practitioner, except those living in a nursing home (12). We defined the entry date in this cohort for the UCORBIO individual with non-obstructed coronary arteries as the day of coronary angiography. For every individual with non-obstructed coronary arteries we selected a matched individual registered in the JGPN who did not have symptoms on the entry date of the individual with non-obstructed coronary arteries. The women and men with non-obstructed coronary arteries were matched to the asymptomatic JGPN individuals on age (in 5-years strata), sex, and general practitioner (1:1). Because a general practitioner's practice has on average 2,500 people enlisted, asymptomatic individuals within these strict selection criteria were limited. Furthermore, as every unique individual could only be selected once, for three individuals with non-obstructed coronary arteries an adequate asymptomatic individual was not available, leaving 136 (98%) matched pairs.

### Data Description

All contacts between enlisted patients and their general practitioner were extracted from the general practitioner's electronical medical file consisting of all primary care consultations. The general practitioner classifies a diagnosis and/or main symptom according to the International Classification for Primary Care (ICPC)–coding system (13) and labels every contact according to this ICPC–coding system. Information on primary care use was collected similar for both women and men with non-obstructed coronary arteries as asymptomatic individuals. We restricted the extracted information to two domains; (suspected) cardiovascular disease and psychosocial problems, either labeled with (“K”) in the ICPC-coding system in case of cardiovascular and (“P”) for psychosocial problems. For the “K” diagnoses and a single “R” code, we further sub-analyzed these for chest pain or pressure (K01, K02), angina pectoris (K74), ischemic heart disease (IHD) (K75 and K76), shortness of breath (R02) and heart failure with preserved or reduced ejection fraction (K77). For some analyses we combined chest pain or pressure (K01, K02), angina pectoris (K74), ischemic heart disease (IHD) (K75 and K76), and shortness of breath (R02) to a single combined variable “angina (equivalent).” As the per-complaint analyses only focused on the most prevalent “K” diagnoses as mentioned above (K01, K02, K74, K75, K76, K77) and not on all possible “K” ICPC codes, the complete “cardiovascular disorders” count will include more complaints and therefore be higher than the sum of the consultations for selected ICPC codes; if an individual consulted the general practitioner for palpitations, this consultation is counted in the “cardiovascular disorders” count but not in the per-complaint analyses. Furthermore, we counted per ICPC code whether there was “at least one” contact with the general practitioner as described above, but did not investigate the number of contacts per ICPC code. We evaluated all complaints separately and per complaint; if an individual consulted the general practitioner for both chest discomfort and shortness of breath during the follow-up period both were counted in the per-complaints analyses. Therefore, the total number of consultations can be higher than the number of participants analyzed in this study.

### Statistical Analyses

Continuous variables that were normally distributed were reported as means with standard deviation, whereas categorical variables were expressed as numbers and percentages. The pairwise McNemar's test was used for the comparison of proportions for cardiovascular or psychosocial consultations between women and men with non-obstructed coronary arteries and asymptomatic individuals. Sex differences were assessed by performing stratified analyses. A *P*-value < 0.05 was considered statistically significant. All statistical analyses were performed using R studio, version 3.5.2 (www.r-project.org).

## Results

### Baseline Characteristics of Women and Men With Non-Obstructed Coronary Arteries

Non-obstructed coronary arteries were more common in women than in men that underwent coronary angiography in the UCORBIO study (39% women and 23% in men). Within the non-obstructed coronary artery population, the total proportion of women and men was 39 vs. 61%. See Figure 1. Baseline characteristics of the study population of women and men with non-obstructed coronary arteries are presented in Table 1. On average, these women were 3 years older than men with non-obstructed coronary arteries. Furthermore, comorbidities and risk factors are prevalent in both women and men (See Table 1). Information on medication use can be found in Supplementary Table 2. Women were more likely non-smoking, to have hypertension at baseline and to be diagnosed with type 2 diabetes than men. However, these differences were not statistically significant. The prevalence of other established risk factors such was comparable between women and men with non-obstructed coronary arteries. In men, even though not statistically significant, a history of myocardial infarction was more often documented than in women. The indication to perform coronary angiography as documented by the cardiologist was most often stable coronary artery disease in both sexes. In both sexes, a non-significant obstruction in one of the epicardial coronaries was more common than no obstruction at all (Table 1).

**Table 1:** Baseline characteristics of women and men with non-obstructed coronary arteries.

**Characteristics**	**Women *N* = 53**	**Men *N* = 86**	***p*-value**
Mean age (SD) – years	66.2 (12.7)	62.5 (11.6)	0.08
Mean body mass index (SD)	26.7 (5.8)	26.6 (4.5)	
Smoking, *n* (%)			0.065
Non smoker	37 (74.0)	43 (53.8)	
Former smoker	8 (16.0)	25 (31.2)	
Active smoker	5 (10.0)	12 (15.0)	
Hypertension, *n* (%)	32 (60.4)	44 (51.2)	0.376
Diabetes, *n* (%)	12 (22.6)	14 (16.3)	0.477
Hypercholesterolaemia, *n* (%)	22 (41.5)	39 (45.3)	0.789
Reduced kidney function, *n* (%)	1 (1.9)	2 (2.3)	1.000
Chest pain, *n* (%)	25 (64.1)	30 (50.0)	0.241
Shortness of breath, *n* (%)	23 (59.0)	20 (33.3)	0.021
LVEF <50%, *n* (%)	3 (6.8)	11 (17.7)	0.178
COPD, *n* (%)	5 (9.4)	6 (7.0)	0.843
Previous MI, *n* (%)	7 (13.2)	22 (25.6)	0.126
Indication for CAG, *n* (%)			
UAP	7 (13.2)	11 (12.8)	0.872
Acute myocardial infarction	4 (7.5)	4 (4.7)	
Stable CAD	30 (56.6)	48 (55.8)	
Other	12 (22.6)	23 (26.7)	
Outcome CAG, *n* (%)			
No CAD	23 (43.4)	19 (22.1)	
Minor CAD (wall irregularities/coronary obstruction(s) <50%)	30 (56.6)	67 (77.9)	

*LVF, left ventricular ejection fraction; MI, myocardial infarction; UAP, unstable angina pectoris; CAD, coronary artery disease; CAG, coronary angiography; COPD, Chronic Obstructive Pulmonary Disease*.

### Cardiovascular Consultations After Coronary Angiography Showed Non-obstructed Coronary Arteries

During a median follow- up of 7 years [IQR 6.4–8.0], 123 (89%) of the women and men with non-obstructed coronary arteries consulted their general practitioner. These consultations (once or more) were labeled for a cardiovascular disorder following the coronary angiography. The consultations of the women and men with non-obstructed coronary arteries were significantly more often [*n* = 123 (89)] than of the asymptomatic individuals (*n* = 46 (34%), *p* < 0.001). In 57% of the cases the consultation was documented for angina or an angina equivalent (Table 2). There were no significant differences between women and men. The general practitioner was consulted by 62% of women and men with non-obstructed coronary arteries within the 1st year of follow up (68% of women and 58% of men). A detailed analysis of the time between the coronary angiography and the cardiovascular and psychosocial consultations in the years following the coronary angiography can be found in Supplementary Figure 1.

**Table 2:** Cardiovascular consultations in women and men with non-obstructed coronary arteries vs. asymptomatic matched individuals.

	**Non-obstructed coronaries** ***N* = 139**	**Asymptomatic matched individuals** ***N* = 136**	***P*-value**
GP consultation for	123 (88.5)	46 (33.8)	<0.001
Cardiovascular disorders, *n* (%)			
*No. of cardiovascular visits, median [IQR]*	17 [5–52]	0 [0–8]	<0.001
Angina (equivalent), *n* (%)	79 (56.8)	15 (11.0)	<0.001
Chest discomfort, *n* (%)	24 (17.3)	1 (0.7)	<0.001
Angina pectoris, *n* (%)	32 (23.0)	3 (2.2)	<0.001
IHD consult, *n* (%)	25 (18.0)	6 (4.4)	<0.001
Shortness of breath, *n* (%)	17 (12.2)	5 (3.7)	0.010
Heart failure, *n* (%)	14 (10.1)	3 (2.2)	0.015

*GP, general practitioner; CAD, coronary artery disease; IHD, ischemic heart disease*.

### Psychosocial Consultations

The label “P” of the ICPC-coding system was used to study the incidence of primary care visits for psychosocial problems. Of the women and men with non-obstructive coronary arteries, 43% were documented under this label as compared to 21 of the asymptomatic individuals (31 vs. 15%, *p* = 0.005). In more detail, these consultations were mainly for anxiety, sleep or mood-related symptoms such as depression (Table 3). This higher prevalence of primary care consultations for psychosocial symptoms in the women and men with non-obstructed coronary arteries as compared with the asymptomatic individuals could not be explained by a higher prevalence of a history of psychological complaints (data not shown).

**Table 3:** Psychological consultations in women and men with non-obstructed coronary arteries vs. asymptomatic matched individuals.

	**Non-obstructed coronaries** ***N* = 139**	**Asymptomatic matched individuals** ***N* = 136**	***P*-value**
GP consultation for	43 (30.9)	21 (15.4)	0.005
Psychological disorder, *n* (%)			
Anxiety, *n* (%)	10 (7.2)	6 (4.4)	
Stress or work-related problems, *n* (%)	2 (1.4)	1 (0.7)	
Sleep disorder, *n* (%)	20 (14.4)	11 (8.1)	
Low mood, *n* (%)	21 (15.1)	6 (4.4)	
Depressive symptoms, *n* (%)	13 (9.4)	2 (1.5)	
Burn out, *n* (%)	6 (4.3)	2 (1.5)	

*GP, general practitioner*.

### Subgroup-Analyses for Men and Women

When we repeated the analyses for women and men separately, similar results for both sexes were found (Table 4).

**Table 4:** Cardiovascular consultations in women and men with non-obstructed coronary arteries vs. asymptomatic individuals.

	**Non-obstructed coronaries**	**Asymptomatic matched individuals**	***P*-value**
**Women**			
GP consultation for	***N*** **= 53**	***N*** **= 52**	
Cardiovascular disorder *n* (%)	48 (90.6)	17 (32.7)	<0.001
Angina(equivalent) *n* (%)	31 (58.5)	6 (11.5)	<0.001
Chest discomfort *n* (%)	8 (15.1)	1 (1.9)	
Angina pectoris *n* (%)	16 (30.2)	1 (1.9)	
IHD *n* (%)	7 (13.2)	1 (1.9)	
Shortness of breath *n* (%)	8 (15.1)	2 (3.8)	
Heart failure *n* (%)	7 (13.2)	2 (3.8)	
**Men**			
GP consultation for	***N*** **= 86**	***N*** **= 84**	
Cardiovascular disorder *n* (%)	75 (87.2)	29 (34.5)	<0.001
Angina(equivalent) *n* (%)	48 (55.8)	9 (10.7)	<0.001
Chest discomfort *n* (%)	16 (18.6)	0 (0.0)	
Angina pectoris n (%)	16 (18.6)	2 (2.4)	
IHD *n* (%)	18 (20.9)	5 (6.0)	
Shortness of breath *n* (%)	9 (10.5)	3 (3.6)	
Heart failure *n* (%)	7 (8.1)	1 (1.2)	

*GP, general practitioner; IHD, ischemic heart disease*.

### Timing of General Practitioner Consultations in WOmen and Men With Non-obstructed Coronaries

**Figure 2:**
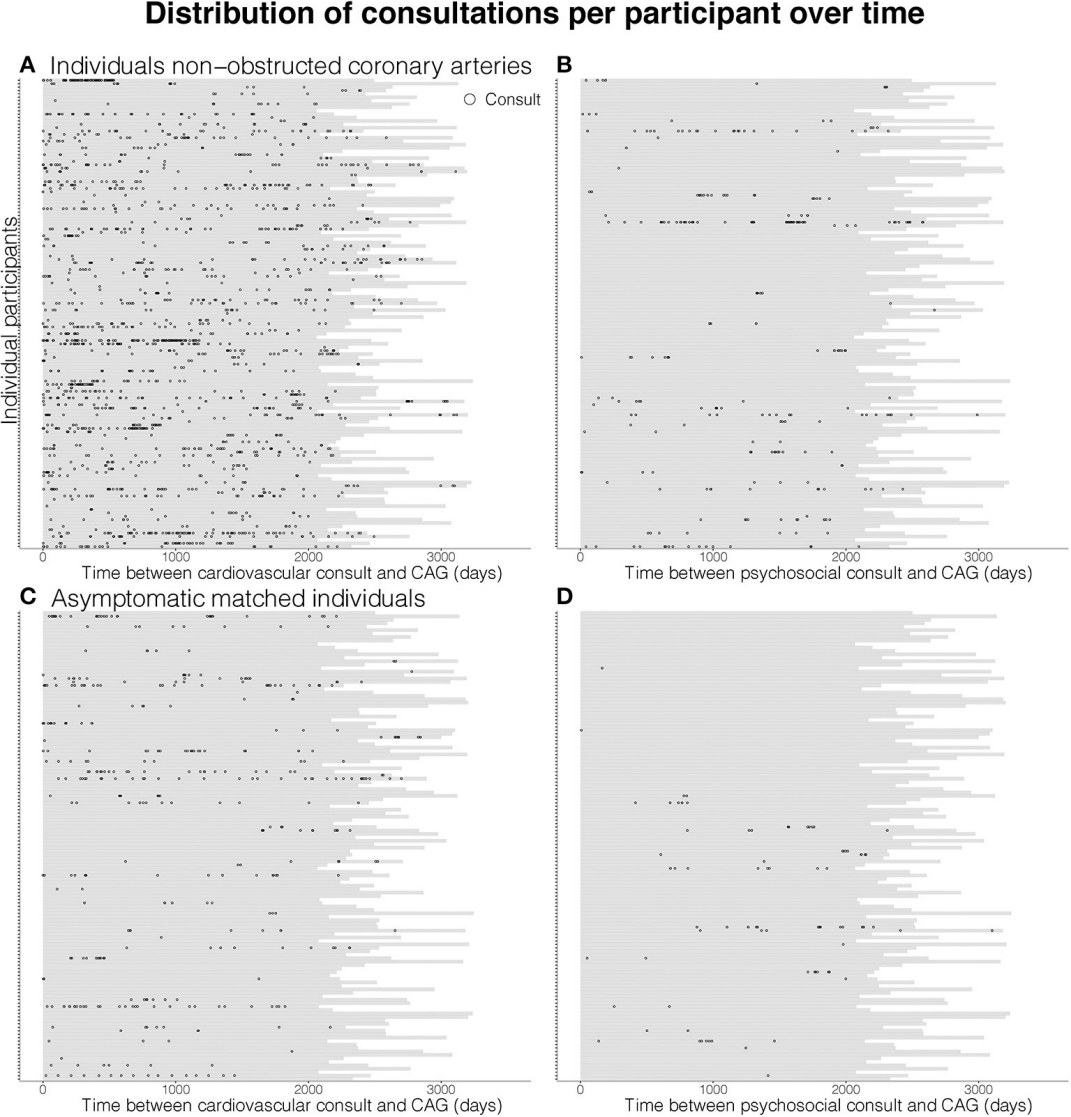
Visualization of cardiovascular (left) and psychological (right) consultations during follow-up after coronary angiography in women and men with non-obstructed coronary arteries (panel **A**, respectively, **B**) and matched asymptomatic men and women (panel **C**, respectively, **D**). Each horizontal bar represents the follow-up period in days of one individual participant and each dot represents a general practitioner consultation.

There is no clear pattern in the timing of the cardiovascular consultations of women and men with non-obstructed coronary arteries (Figure 2). Yet, the difference in the number of consultations for cardiovascular and psychosocial consultations during follow-up between individuals with non-obstructed coronary arteries (Figure 2, panel **A**, respectively, panel **B**) and asymptomatic matched individuals (Figure 2, panel **C**, respectively, panel **D**) is striking. Detailed timing of consultations is documented in Supplementary Figure 1.

## Discussion

Our study shows that the majority of both women and men with non-obstructed coronary arteries remain symptomatic in the years following the coronary angiography. They consult their general practitioner more often for cardiovascular and psychosocial care than matched asymptomatic individuals. Although the prevalence of non-obstructed coronary arteries is higher among women (6), our data does not indicate that there are differences between women and men in health needs in the years following coronary angiography. Notably, angina equivalent symptoms were more seen, but also a high burden of consultations for psychosocial symptoms was recorded, as compared with asymptomatic women and men.

Previous studies highlighted that individuals with non-obstructed coronary arteries were not as healthy as previously thought (11, 14, 15). Our results support these findings, as up to 90% of these individuals remain symptomatic, are likely to experience psychological issues, and pursue medical care for these complaints, mostly at the general practitioner.

Although the absolute numbers are small, women and men with non-obstructed coronary arteries seem more prone to developing heart failure. This has been described before and a likely pathophysiological explanation for this phenomenon might be underlying microvascular disease (2). While the prevalence of microvascular disease has shown to be high in symptomatic women without obstructive coronary artery disease (15), our data do not indicate that men have less symptoms of heart failure in the years following angiography. Another possible explanation could be epicardial vasospasm, which is not regularly assessed during coronary angiography but is known to be a frequent cause of anginal complaints in the absence of obstructive coronary artery disease (3, 4). Yet, vasospastic angina as an isolated pathology does not seem to be accompanied by a poor prognosis (16).

In addition to cardiovascular complaints, the proportion of women and men that consult their general practitioner for psychological complaints is substantial. Although we were unable to assess causality due to the observational nature of this study, the association between psychosocial burden and non-obstructed coronary arteries seems to be strong in both women and men. These results are in line with previous research showing depression to be related to both the development as well as adverse outcomes of coronary artery disease (17). Even though previous research suggests the risk might be higher in females as compared to males (18, 19), we do not find evidence for this in our study. However, due to limited power, we cannot exclude or confirm sex differences in psychological complaints in these patients. The high burden of psychological complaints in this specific group of patients warrants urgent attention, both in clinical care as well as in research.

It is established that women and men with non-obstructed coronary arteries and prolonged anginal symptoms are at risk for (other) cardiovascular diseases, and with a rather poor prognosis (1). To date, it was unknown how large the population “at risk” is. This study has provided evidence that the number of patients with prolonged or recurrent symptoms suggestive of cardiac pathology is high in both sexes.

Our results suggest that the healthcare burden and costs are substantial, due to repeated general practitioner visits, the possible additional diagnostic tests that are performed and the economic burden, given that the majority of this population is still at working age. In the WISE study the costs of symptom-driven care in women with persistent angina was reported to be almost 750.000 dollars per year (10). Even though healthcare costs cannot be compared between countries, it does provide insight in the extensive costs that are associated with symptomatic women and men with non-obstructed coronary arteries. Based on nation-wide reports, on average 20% of the patients who consult the cardiologist for chest discomfort the first time, at least a functional or anatomical investigation is performed, including over 10.000 coronary angiographies each year (17). Extrapolating our results, in 27% of these coronary angiographies non-obstructed coronary arteries would be the result of whom 90% (i.e., *n* > 2,000), would return to the general practitioner with prolonged complaints.

## Strengths and Limitations

The strengths of this study are the fact that we have compared groups of the same source population and we included both women and men. Also, as we evaluated all women and men in whom non-obstructed coronary arteries were found, instead of selecting only those with prolonged complaints, we could evaluate the magnitude of the proportion of women and men with non-obstructed coronary arteries that remained symptomatic. Furthermore, we studied the health needs for both cardiovascular and psychological issues for a median duration of seven years. This study also has a number of limitations. Our study population did not undergo further coronary function assessment to assess whether microvascular disease or vasospasm was the underlying cause; extensive invasive testing protocols on women and men suffering from ischemia with non-obstructed coronary arteries have been recently established by the coronary vasomotor working group (COVADIS) (18), guiding when and how to evaluate coronary vascular dysfunction or vasospasm in these patients. These additional tests are currently being implemented in clinical care. Randomized trials assessing feasibility and value of these tests in improvement of quality of life and angina in these women and men (19) show promising results, possible due to improved perception of disease and/or tailored medical treatment. As the data was collected during regular care, we only had access to the data as documented by the general practitioner. Thus, we had to rely on International Classification for Primary Care codes to determine the prevalence of complaints. However, since general practitioners in the Netherlands are trained to only allocate a specific code to a patient if they are reasonably sure of their diagnosis, these codes can be used with a reasonably high certainty (20). Only 20% of the eligible women and men with non-obstructed coronary arteries in the UCORBIO database could be identified within the JGPN database. This selection was “at random”; as the women and men identified within the JGPN did not differ from the not-linked UCORBIO women and men, we can exclude selection bias. Yet, the power to perform detailed subgroup analyses was low and we could only compare the most prevalent complaints sex-stratified. Finally, as we performed our study in one center with a relatively homogeneous Caucasian population, our results might not be generalizable to other ethnic populations undergoing coronary angiography.

## Conclusions

The diagnosis of non-obstructed coronary arteries is more common in women than in men undergoing coronary angiography. The majority of the angina population without obstructive CAD remains symptomatic following coronary angiography. They had over 2-fold higher health needs for angina, heart failure, and psychosocial complaints compared to controls. There were no differences between men and women's health needs following coronary angiography without obstructive CAD.

## Data Availability Statement

The raw data supporting the conclusions of this article will be made available by the authors, without undue reservation.

## Ethics Statement

The studies involving human participants were reviewed and approved by Medical Research Ethics Committee of the University Medical Center Utrecht. The patients/participants provided their written informed consent to participate in this study.

## Author Contributions

FG undertook the writing of the paper, developed the analysis plan, and analyzed the data. AE guided the writing of the paper and the statistical analysis of the results. ZR and CG assisted the interpretation of the data and made substantial improvements to the paper. IH, GP, and FA made substantial improvements to the paper. FR guided the statistical analysis of the results and made substantial improvements to the paper. HD and NO-M supervised the study, contributed to the data analysis plan, and made substantial improvements to the paper. All authors have read and given final approval of the submitted manuscript.

## Conflict of Interest

The authors declare that the research was conducted in the absence of any commercial or financial relationships that could be construed as a potential conflict of interest.
