# Supplementary material for: Persistent Symptoms and Health Needs of Women and Men With Non-Obstructed Coronary Arteries in the Years Following Coronary Angiography
Source: Front Cardiovasc Med. 2021 May 3;8:670843. doi: 10.3389/fcvm.2021.670843 (PMC8126611; doi:10.3389/fcvm.2021.670843)
Supplement: Supplementary file 1 [file Image_1.PDF]

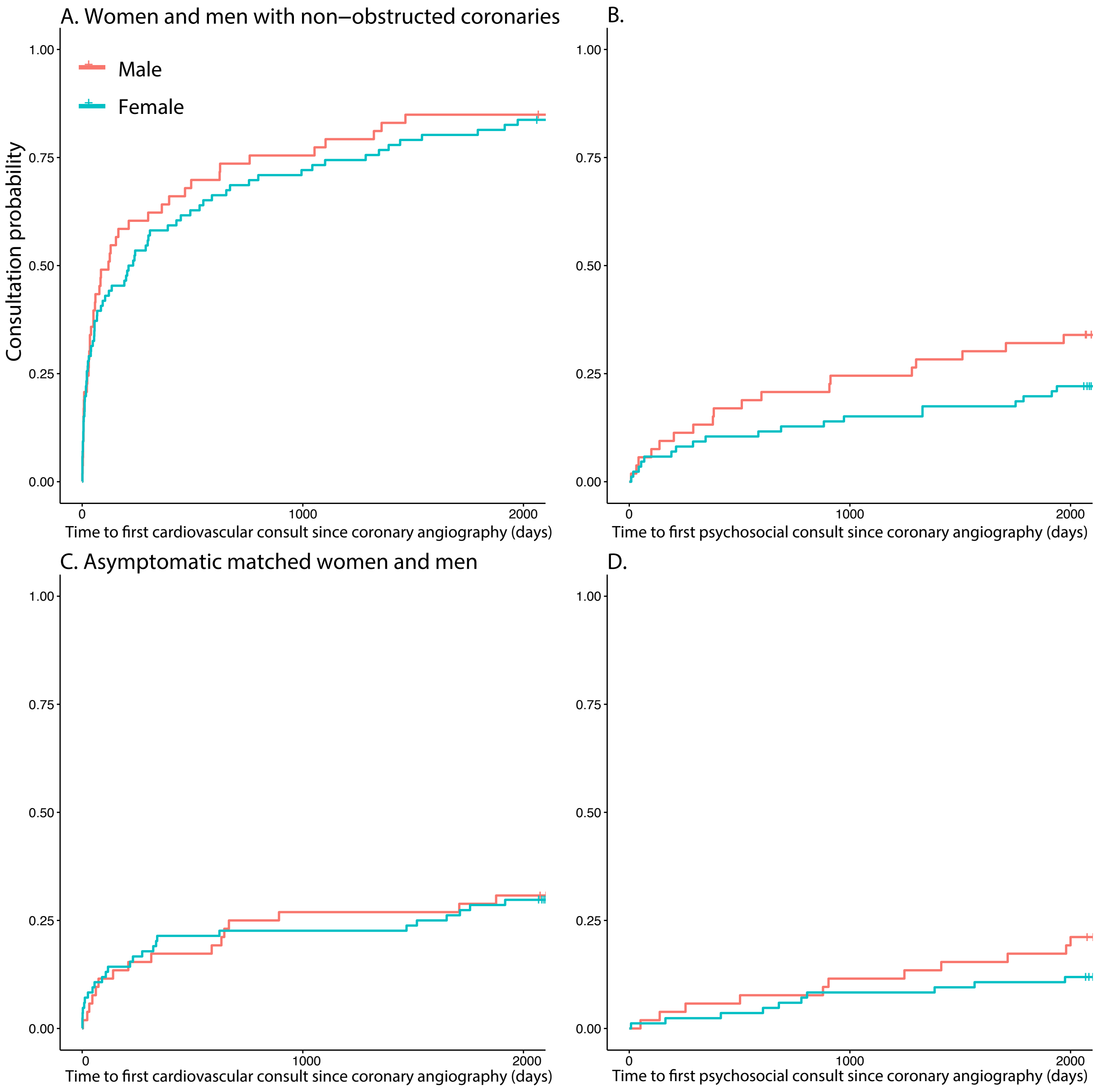


**Supplementary Figure 1. Timing of first cardiovascular (left) and psychological (right) consultations at the general practitioner for women and men with non-obstructed coronaries (above) following coronary angiography and matched individuals (below).**
