# Supplementary material for: Persistent Symptoms and Health Needs of Women and Men With Non-Obstructed Coronary Arteries in the Years Following Coronary Angiography
Source: Front Cardiovasc Med. 2021 May 3;8:670843. doi: 10.3389/fcvm.2021.670843 (PMC8126611; doi:10.3389/fcvm.2021.670843)
Supplement: Supplementary file 2 [file Table_1.docx]

**Supplementary table 1. Comparison of UCORBIO men and women with non-obstructed coronary arteries that could be identified in the JGPN database compared with those that could not be identified and therefore were not included in this study.**

|  | Overall | No JGPN | JGPN |
| --- | --- | --- | --- |
| n | 687 | 548 | 139 |
| Women, n (%) | 267 (38.9) | 214 (39.1) | 53 (38.1) |
| Mean age (SD) – years | 62.05 (12.06) | 61.57 (12.00) | 63.92 (12.15) |
| Smoking, n (%) |  |  |  |
| Non smoker | 385 (59.7) | 305 (59.2) | 80 (61.5) |
| Former smoker | 158 (24.5) | 125 (24.3) | 33 (25.4) |
| Active smoker | 102 (15.8) | 85 (16.5) | 17 (13.1) |
| Hypertension, n (%) | 359 (52.3) | 283 (51.6) | 76 (54.7) |
| Diabetes, n (%) | 112 (16.3) | 86 (15.7) | 26 (18.7) |
| Hypercholesterolemia, n(%) | 287 (41.8) | 226 (41.2) | 61 (43.9) |
| Mean body mass index (SD)) | 26.2 (4.7) | 26.1 (4.7) | 26.6 (5.0) |
| Kidney disease, n (%) | 23 ( 3.3) | 20 ( 3.6) | 3 ( 2.2) |
| Chest pain, n (%) | 239 (43.9) | 184 (41.3) | 55 (55.6) |
| Shortness of breath, n (%) | 187 (34.3) | 144 (32.3) | 43 (43.4) |
| LVEF <50%, n (%) | 105 (18.5) | 91 (19.7) | 14 (13.2) |
| COPD, n (%) | 60 ( 8.7) | 49 ( 8.9) | 11 ( 7.9) |
| Previous MI, n (%) | 119 (17.3) | 90 (16.4) | 29 (20.9) |
| Indication, n (%) |  |  |  |
| UAP | 49 ( 7.1) | 31 ( 5.7) | 18 (12.9) |
| Myocardial infarction | 41 ( 6.0) | 33 ( 6.0) | 8 ( 5.8) |
| Other | 194 (28.2) | 159 (29.0) | 35 (25.2) |
| Stable CAD | 403 (58.7) | 325 (59.3) | 78 (56.1) |
| Minor CAD (%)  (wall irregularities/coronary obstruction(s) <50%) | 464 (67.5) | 367 (67.0) | 97 (69.8) |

Abbreviations: LVF, left ventricular ejection fraction; MI, myocardial infarction; UAP, unstable angina pectoris; CAD, coronary artery disease; CAG, coronary angiography.
