# Supplementary material for: Persistent Symptoms and Health Needs of Women and Men With Non-Obstructed Coronary Arteries in the Years Following Coronary Angiography
Source: Front Cardiovasc Med. 2021 May 3;8:670843. doi: 10.3389/fcvm.2021.670843 (PMC8126611; doi:10.3389/fcvm.2021.670843)
Supplement: Supplementary file 3 [file Table_2.docx]

**Supplementary table 2. Baseline medication use of women and men with non-obstructed coronary arteries stratified by sex.**

|  | Women | Men |
| --- | --- | --- |
| n | 53 | 86 |
| Aspirin use = yes, n (%) | 29 (54.7) | 44 (51.2) |
| Clopidogrel use = yes, n (%) | 2 ( 3.8) | 10 (11.6) |
| Betablocker use = yes, n (%) | 25 (47.2) | 41 (47.7) |
| Calcium antagonist use = yes, n (%) | 19 (35.8) | 20 (23.3) |
| Nitrates use = yes, n (%) | 7 (13.2) | 7 ( 8.1) |
| Nitroglycerin use = yes, n (%) | 8 (15.1) | 16 (18.6) |
| ACE-inhibitor use = yes, n (%) | 19 (35.8) | 30 (34.9) |
| ARB use = yes, n (%) | 6 (11.3) | 9 (10.5) |
| Diuretic use = yes, n(%) | 20 (37.7) | 23 (26.7) |
| Statin use = yes, n (%) | 26 (49.1) | 44 (51.2) |
| Ezetimibe use = yes, n (%) | 6 (11.3) | 3 ( 3.5) |
| Oral anti-diabetic medications use = yes, n (%) | 11 (20.8) | 12 (14.0) |

Abbreviations: ACE, angiotensin converting enzyme; ARB, angiotensin receptor blocker
